# Guidelines for morpholino use in zebrafish

**DOI:** 10.1371/journal.pgen.1007000

**Published:** 2017-10-19

**Authors:** Didier Y. R. Stainier, Erez Raz, Nathan D. Lawson, Stephen C. Ekker, Rebecca D. Burdine, Judith S. Eisen, Philip W. Ingham, Stefan Schulte-Merker, Deborah Yelon, Brant M. Weinstein, Mary C. Mullins, Stephen W. Wilson, Lalita Ramakrishnan, Sharon L. Amacher, Stephan C. F. Neuhauss, Anming Meng, Naoki Mochizuki, Pertti Panula, Cecilia B. Moens

**Affiliations:** 1 Department of Developmental Genetics, Max Planck Institute for Heart and Lung Research, Bad Nauheim, Germany; 2 Institute of Cell Biology, ZBME, University of Münster, Münster, Germany; 3 Department of Molecular, Cell, and Cancer Biology, University of Massachusetts Medical School, Worcester, Massachusetts, United States of America; 4 Mayo Clinic, Rochester, Minnesota, United States of America; 5 Department of Molecular Biology, Princeton University, Princeton, New Jersey, United States of America; 6 Institute of Neuroscience, University of Oregon, Eugene, Oregon, United States of America; 7 Lee Kong Chian School of Medicine, Nanyang Technological University, Singapore; 8 The Living Systems Institute, University of Exeter, Exeter, United Kingdom; 9 Institute of Cardiovascular Organogenesis and Regeneration, WWU Münster, Faculty of Medicine, Münster, Germany; 10 Division of Biological Sciences, University of California, San Diego, La Jolla, California, United States of America; 11 Division of Developmental Biology, NICHD, NIH, Bethesda, Maryland, United States of America; 12 Department of Cell and Developmental Biology, University of Pennsylvania Perelman School of Medicine, Philadelphia, Pennsylvania, United States of America; 13 Department of Cell and Developmental Biology, University College London, London, United Kingdom; 14 Molecular Immunity Unit, Department of Medicine, University of Cambridge, MRC Laboratory of Molecular Biology, Cambridge, United Kingdom; 15 Departments of Molecular Genetics and Biological Chemistry and Pharmacology, Ohio State University, Columbus, Ohio, United States of America; 16 Institute of Molecular Life Sciences, University of Zurich, Zurich, Switzerland; 17 School of Life Sciences, Tsinghua University, Beijing, China; 18 National Cerebral and Cardiovascular Center Research Institute, Osaka, Japan; 19 Department of Anatomy and Neuroscience Center, University of Helsinki, Helsinki, Finland; 20 Division of Basic Sciences, Fred Hutchinson Cancer Research Center, Seattle, Washington, United States of America; Stanford University School of Medicine, UNITED STATES

The zebrafish (*Danio rerio*) has emerged as a powerful model to study vertebrate development and disease. Its short generation time makes it amenable to genetic manipulation and analysis, and its small size and high fecundity make it especially well suited for large-scale forward genetic and chemical screens. Fast-developing zebrafish embryos are transparent, facilitating live imaging of a variety of developmental processes in wild-type and mutant animals.

The zebrafish was originally chosen as a model with forward genetics in mind and has been used successfully in countless genetic screens. However, it quickly became a priority to be able to disrupt the function of specific genes in a targeted way. In the early 2000s, in the absence of tools for efficient targeted mutagenesis, morpholino (MO) antisense oligomers became the tool of choice for gene knockdown in a range of models including *Xenopus*, zebrafish, sea urchin, and chick [1, 2]. MOs are chemically synthesized oligomers that are typically injected into embryos at the 1-cell stage, bind complementary target mRNAs, and prevent their translation or splicing. They are similar to small interfering RNAs (siRNAs) and short hairpin RNAs (shRNAs) in that they interfere with the function of a gene without altering its sequence.

Based on their ability to phenocopy well-characterized mutants, MOs were rapidly adopted by zebrafish researchers to disrupt the function of genes that had not been uncovered by forward genetic screens. However, concerns soon arose about the off-target effects of MOs, motivating leading scientists in the zebrafish and *Xenopus* fields to publish a set of guidelines including controls and rescue experiments that, if followed carefully, would help distinguish specific phenotypes from off-target effects [3]. Since that time, the number of zebrafish lines carrying mutations in genes of interest has grown, at first gradually, from ongoing forward genetic screens and TILLING efforts, and then exponentially, with the advent of zinc finger nucleases, TALENs, and CRISPRs. The ease of generating mutants now allows the reevaluation of MO-induced versus mutant phenotypes and has led to the surprising finding that, even with the careful application of the Eisen and Smith guidelines [3], MO-induced phenotypes can be different and often more severe than those of the corresponding mutants.

This brief document provides an updated set of guidelines regarding the use of MOs in zebrafish that we anticipate will be of value for experimentalists as well as journal and grant reviewers, and decision makers. These general guidelines build upon those by Eisen and Smith [3] taking into account new developments and findings in the field, namely, the following:

1. In addition to the wealth of available ENU and insertional mutant alleles in zebrafish, TALEN and CRISPR/Cas9 tools have made genome editing widely possible;
2. MO-induced phenotypes are often more severe than those of the corresponding mutants, which could be due to the following:
  - phenotypic rescue of zygotic mutants by maternally provided wild-type mRNAs, the translation of which can be blocked by MOs,
  - off-target effects of the MO (e.g., Kok et al. 2015 [4]),
  - the hypomorphic nature of the mutant allele analyzed, or
  - genetic compensation in mutant but not MO-injected animals (also known as morphants) (e.g., Rossi et al. 2015 [5]).

MOs are like any other knockdown reagents; their use should thus be evaluated by journal/grant reviewers and decision makers accordingly (i.e., on a comparable footing to siRNAs, for example). However, antisense approaches are not a form of reverse genetics.

As mentioned above, the advent of TALEN and CRISPR/Cas9 tools has now made it routine to generate stable mutant lines and hence to use reverse genetics to study gene function in zebrafish. Additionally, mutant alleles for many genes are now readily available through zebrafish community resource centers. Thus, MOs should be used alongside mutant(s) for the corresponding gene. When used in line with the following guidelines, MO-based studies can complement mutant studies, providing an additional and useful approach to our understanding of gene function.

**Fig 1:**
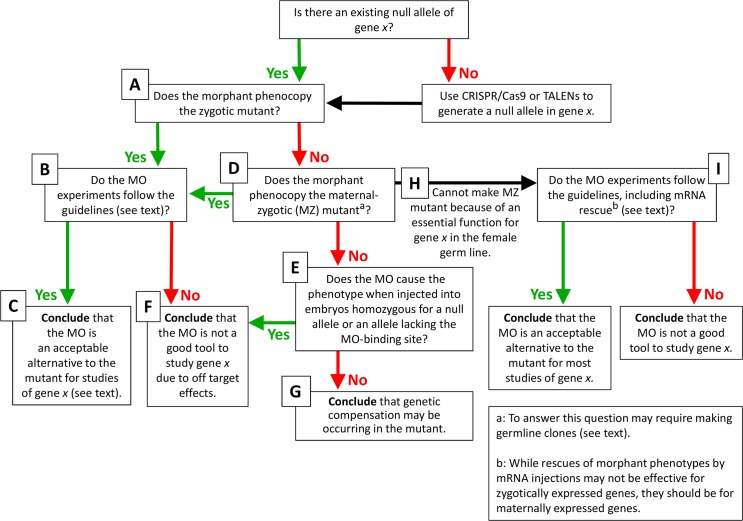
Flowchart for zebrafish researchers interested in using morpholinos (MOs) to study the function of gene *x*.

The possibility of obtaining or generating mutants allows the morphant and mutant phenotypes to be directly compared (Fig 1A). If they are the same, and if the MO experiments satisfy the criteria initially laid out by Eisen and Smith [3] (Fig 1B and see below), then the morphant can be considered an acceptable alternative to the mutant for follow-up studies concerning that specific phenotype (e.g., generating a partial loss-of-function series by using progressively lower doses of MO, or generating a population of embryos knocked down for a specific gene product, Fig 1C). (Note that caution should be exercised when conducting molecular analyses [e.g., transcriptome or proteome], as it is likely that MOs cause additional effects besides blocking the translation/splicing of target mRNAs.) If a new phenotype is analyzed or the same phenotype examined using a different assay, the mutants and morphants should first be compared directly before considering the morpholino validated.

If a morphant phenotype is more severe than, or different from, a mutant phenotype, decisive experiments can be performed to distinguish between the 4 possible reasons listed above: (i) To test the possibility that the morphant phenotype is more severe because the zygotic mutant is rescued by maternally provided wild-type mRNAs, the morphant phenotype can be compared to the maternal-zygotic (MZ) mutant phenotype (Fig 1D). (Note that, for maternally expressed genes with essential zygotic functions, the generation of viable and fertile homozygous mutant females is not possible. In such cases, wild-type females with homozygous mutant germ lines can be generated by germ line replacement [6]. If, however, the gene of interest has an essential cell-autonomous function in the development of the germ line, germ line replacement is not possible and thus one will not be able to directly compare the morphant and MZ mutant phenotypes; in such cases, given the current technology, gene function can only be studied using nongenetic tools (Fig 1H).) (ii) A decisive approach to determine the optimal sequence and dose of a MO that does not cause off-target effects is to inject the MO into embryos whose genome (and whose mother’s genome, for maternally expressed genes) has been edited so as to eliminate the MO-binding site or to eliminate the function of the target gene (Fig 1E). (Note that demonstrating that an allele is a null can be challenging either genetically, as very few deficiency alleles are available in zebrafish, or biochemically, as antibodies recognizing various epitopes along the protein are usually not available. Whole-gene deletions might also affect additional genetic elements present in introns, and recent studies have revealed a surprising ability of the genome to circumvent putatively deleterious nonsense and frameshift mutations [7, 8, 9]. Care should thus be taken when designing targeting tools to avoid these pitfalls.) Any additional phenotype caused by the MO beyond that of the mutant is by definition an off-target effect and invalidates the use of the MO (Fig 1F). If, however, a MO phenotype that is detected in wild-type embryos disappears or is strongly suppressed upon injection into embryos homozygous for a null allele, such an observation suggests that genetic compensation may be occurring in the mutant (Fig 1G).

In summary, a MO should be validated by comparison to a mutant, and if there is a discrepancy, by injection into embryos homozygous for a null allele or an allele lacking the MO-binding site. Although the control of injecting the MO into a mutant provides the most definitive evidence for MO specificity, we suggest the following additional guidelines for MO use, especially for situations in which a mutant cannot be generated.

- Multiple MOs (ATG and splice blocking), or MOs and another approach (e.g., CRISPR-induced mutagenesis or CRISPR interference [CRISPRi]) should be used to target individual genes, and their efficiency should be assessed whenever possible (e.g., using antibodies, RT-PCR, qPCR) to minimize the amount of MO injected.
- Rescue experiments should be attempted for the approaches listed above (e.g., by injection of mRNA or DNA lacking the MO-binding site in the case of MO studies), and if rescue is successful, control experiments should be conducted (e.g., using mutant RNA or DNA, i.e., RNA or DNA that does not encode a functional gene product).
- An injection control MO (standard negative control MO, 5-base mismatch MO, or a suitable alternative MO) should be used to account for developmental delay. Such MOs cannot serve as controls for the specificity of the experimental MO.
- The approach of validating ATG MOs by assessing their ability to suppress the expression of a co-injected target mRNA-GFP fusion is of little value as we now know that suppression of GFP expression is generally observed and it does not test the effect of the MO on the endogenous RNA. This control is no longer recommended
- Essential routine procedures include a dose response curve (extra caution should be exercised when one has to inject more than 5 ng of a MO to cause a phenotype [10]), the examination of statistically meaningful numbers of control and experimental animals, extensive documentation of the penetrance and expressivity of all phenotypes, and the use of blinding strategies whenever possible.

Finally, a word of caution that previous publication of MOs is not a guarantee of their fidelity, particularly if a new phenotype is being described. These reagents should be critically evaluated to ascertain that they were properly characterized; ideally, key controls such as dose response curves and rescue experiments should be repeated when using these tools, and such data included in the manuscript.

Of course, there will be exceptions when the full set of guidelines cannot be followed. In these cases, appropriate caveats should be considered and included in the text that describes the use of the MOs, the results obtained, and their interpretation.

In conclusion, we hope that these brief and mostly conceptual guidelines will assist scientists working with zebrafish as well as those assessing manuscripts and grant proposals based on experiments using zebrafish. In addition, these guidelines may become helpful for other communities using antisense reagents to study gene function.
